# Novel Loss of Function Variant in BCKDK Causes a Treatable Developmental and Epileptic Encephalopathy

**DOI:** 10.3390/ijms23042253

**Published:** 2022-02-18

**Authors:** François Boemer, Claire Josse, Géraldine Luis, Emmanuel Di Valentin, Jérôme Thiry, Christophe Cello, Jean-Hubert Caberg, Caroline Dadoumont, Julie Harvengt, Aimé Lumaka, Vincent Bours, François-Guillaume Debray

**Affiliations:** 1 Biochemical Genetics Laboratory, Department of Human Genetics, CHU of Liege, University of Liege, 4000 Liege, Belgium; Geraldine.Luis@chuliege.be (G.L.); christophe.cello@chuliege.be (C.C.); 2 Department of Medical Oncology, CHU of Liege, University of Liege, 4000 Liege, Belgium; c.josse@chuliege.be (C.J.); j.thiry@uliege.be (J.T.); 3 Laboratory of Human Genetics, Department of Biomedical and Preclinical Sciences, Groupe Interdisciplinaire de Génoprotéomique Appliquée-Recherche (GIGA-R), University of Liege, 4000 Liege, Belgium; aime.lumaka@uliege.be; 4 Viral Vector Platform, Groupe Interdisciplinaire de Génoprotéomique Appliquée-Recherche (GIGA-R), University of Liege, 4000 Liege, Belgium; edivalentin@uliege.be; 5 Molecular Genetics Laboratory, Department of Human Genetics, CHU of Liege, University of Liege, 4000 Liege, Belgium; jh.caberg@chuliege.be; 6 Department of Pediatrics, CHC MontLégia, 4000 Liege, Belgium; caroline.dadoumont@chc.be; 7 Center of Genetics, Department of Human Genetics, CHU of Liege, University of Liege, 4000 Liege, Belgium; julie.harvengt@chuliege.be (J.H.); vbours@uliege.be (V.B.); 8 Metabolic Unit, Department of Human Genetics, CHU of Liege, University of Liege, 4000 Liege, Belgium; fg.debray@chuliege.be

**Keywords:** BCKDK deficiency, branched-chain amino acids, autism, epilepsy, newborn screening

## Abstract

Branched-chain amino acids (BCAA) are essential amino acids playing crucial roles in protein synthesis and brain neurotransmission. Branched-chain ketoacid dehydrogenase (BCKDH), the flux-generating step of BCAA catabolism, is tightly regulated by reversible phosphorylation of its E1α-subunit. BCKDK is the kinase responsible for the phosphorylation-mediated inactivation of BCKDH. In three siblings with severe developmental delays, microcephaly, autism spectrum disorder and epileptic encephalopathy, we identified a new homozygous in-frame deletion (c.999_1001delCAC; p.Thr334del) of *BCKDK*. Plasma and cerebrospinal fluid concentrations of BCAA were markedly reduced. Hyperactivity of BCKDH and over-consumption of BCAA were demonstrated by functional tests in cells transfected with the mutant BCKDK. Treatment with pharmacological doses of BCAA allowed the restoring of BCAA concentrations and greatly improved seizure control. Behavioral and developmental skills of the patients improved to a lesser extent. Importantly, a retrospective review of the newborn screening results allowed the identification of a strong decrease in BCAA concentrations on dried blood spots, suggesting that BCKDK is a new treatable metabolic disorder probably amenable to newborn screening programs.

## 1. Introduction

In humans, branched-chain amino acid (BCAA; leucine, isoleucine and valine) homeostasis is essentially under the control of the mitochondrial branched-chain α-ketoacid dehydrogenase (BCKDH) complex, which catalyzes the oxidative decarboxylation of branched short-chain α-ketoacids [1]. Beside their role of substrate for protein synthesis, BCAA are involved in multiple metabolic pathways and play essential functions in growth and development [2,3,4].

The main inherited disorder related to BCAA homeostasis is maple syrup urine disease (MSUD). Involving either *BCKDHA*, *BCKDHB, DBT* or *DLD* genes, MSUD is characterized by an accumulation of BCAA and branched-chain ketoacids, which can induce fatal acidosis and severe neurological impairment [5]. Besides classical MSUD, deficiencies in *BCAT2* [6], *PPM1K* [7] and *BCKDK* [8] genes have also recently been identified as disruptors of BCAA homeostasis.

The branched-chain α-ketoacid dehydrogenase kinase (BCKDK) enzyme is an important regulator of the catabolic pathways of BCAA. Catalyzing the phosphorylation-mediated inactivation of the E1α-subunit of BCKDH complex, a loss of function of BCKDK enzyme therefore causes an increase in BCAA catabolism. Consequently, patients with BCKDK deficiency present reduced concentrations of BCAA in body fluids. Clinically, the disorder is characterized by developmental delays, intellectual disability, epilepsy and autism spectrum disorder with neurobehavioral abnormalities [8,9].

Here, we report the identification of a novel variant in the *BCKDK* gene identified in three siblings of an inbred family. We describe the experiments we carried out to confirm the pathogenicity of this new variant and present the clinical follow-up of affected patients under treatment.

## 2. Results

### 2.1. Novel Mutation

**Figure 1:**
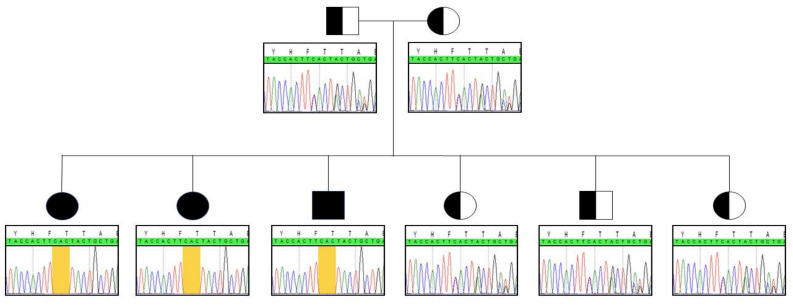
Segregation analysis within the family. Yellow frame indicates the location of the indel.

Whole exome sequencing unveiled the in-frame c.999_1001delCAC (p.Thr334del) homozygous deletion in the *BCKDK* gene in patient 1. Sanger sequencing was subsequently performed to confirm the variant. To the best of our knowledge, this indel has not been previously described. Genotyping the deleterious gene in patients 2 and 3 highlighted the same homozygous anomaly. Both parents and the youngest siblings were all heterozygous carriers of the indel (Figure 1).

### 2.2. Protein Conformation and Loss of Function

**Figure 2:**
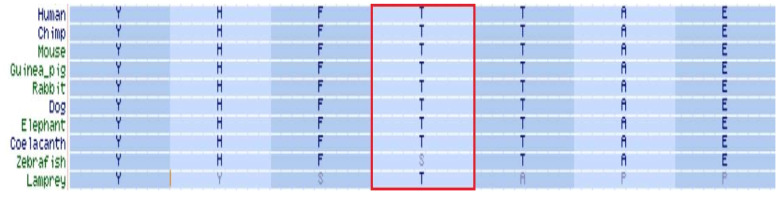
BCKDK orthology. Threonine in position 334 of BCKDK protein is marked by a red frame.

Located within the kinase-activity domain of the BCKDK protein [8,10], the threonine residue in position 334 is highly conserved throughout evolution (Figure 2), suggesting a crucial role in enzymatic activity.

**Figure 3:**
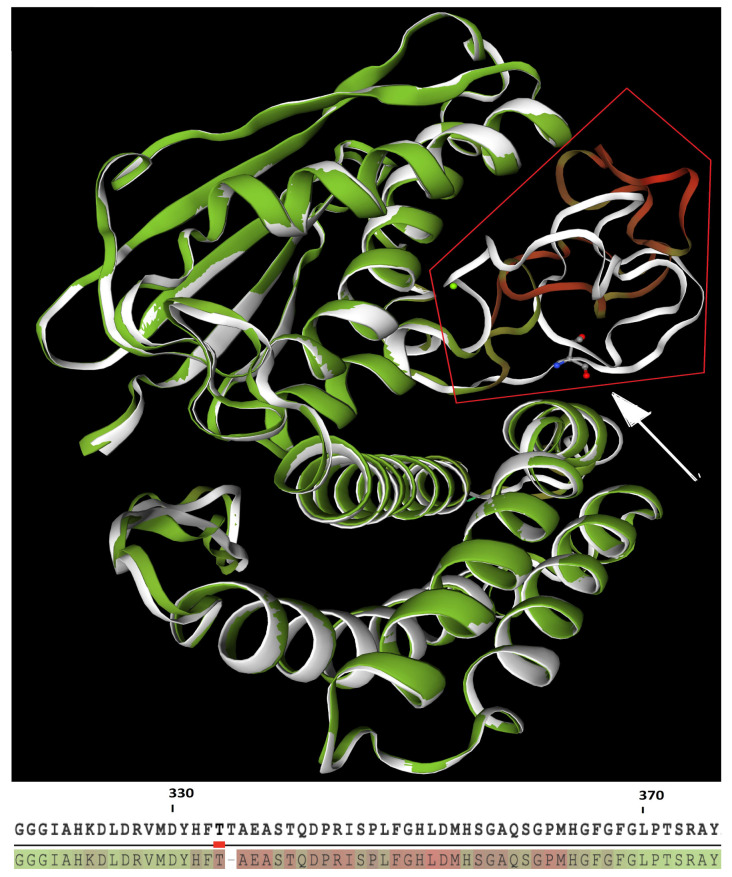
**Upper**: Structural superposition of wild-type (white structure) and mutant (green structure) BCKDK. Red frame highlights the kinase-activity domain, presenting divergent folding between WT and mutant (green/red structure) protein. White arrow points to the deleted threonine of the WT protein. **Lower**: relative consistency between WT (**upper** line) and mutant (**lower** line) proteins, where green residues have the highest consistency scores, while red one have the lowest scores.

According to the LDDT computation, the indel c.999_1001delCAC induces an important remodeling of the kinase-activity domain. The folding difference of this substructure is a consequence of the low relative consistency (i.e., large pairwise interatomic distances) of each residue located between histidine in position 332 and glycine in position 367 (Figure 3).

Severely reduced levels of BCAA were measured in plasma and CSF of affected patients. A retrospective review of newborn screening results in patient 1 revealed very low leucine/isoleucine (Xle) concentration, at 84 μmol/L. Valine was also significantly reduced, at 47 μmol/L. In patient 2 and 3, NBS Xle concentrations were respectively measured at 89 µmol/L and 210 µmol/L, while valine levels were at 44 and 81 μmol/L, respectively. BCAA results of affected patients and their relatives are summarized in Table 1.

**Figure 4:**
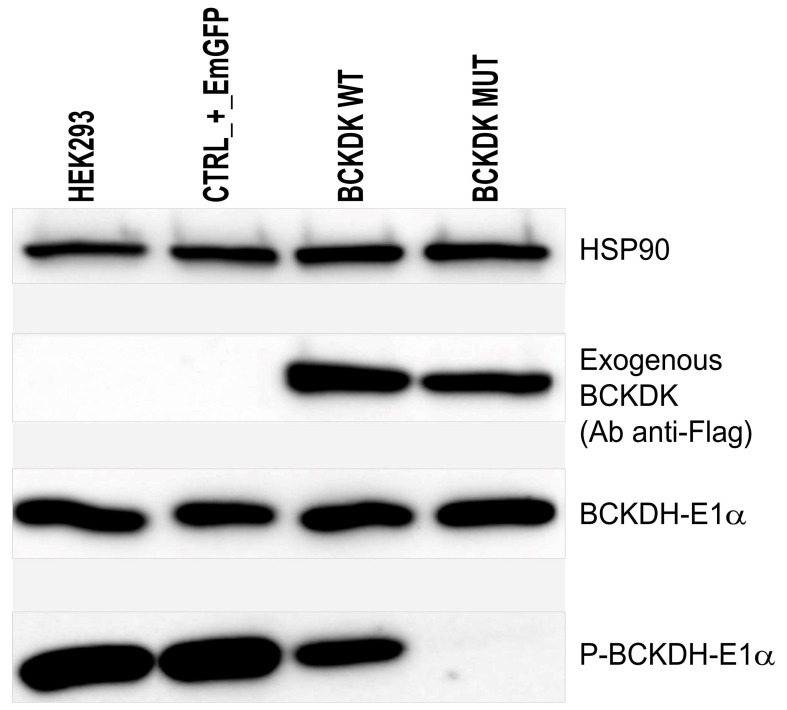
Western blot to detect exogenous BCKDK (Ab anti-Flag); E1α subunit of BCKDH; phosphorylated form of E1α subunit of BCKDH, and HSP90 as charge control. From left to right: HEK-293 were left untreated, transduced with a transduction control (EmGFP), transduced with wild-type exogenous BCKDK, and transduced with mutated form of BCKDK (p.Thr334del).

The loss of BCKDK function caused by the homozygous c.999_1001delCAC indel was finally demonstrated on cultured cells. Lentivirus transduction of HEK-293 cells was performed to over-express an exogenous form of WT or mutated BCKDK (NP_005872.2). Over-expressed exogenous BCKDK sequences included silent mutations, so the endogenous BCKDK protein could be silenced using additional lentivirus containing shRNA. Five different shRNA targeting the endogenous BCKDK mRNA, were tested and three were selected for their relative efficacy to downregulate the endogenous BCKDK expression (Supplementary Figure S1A). However, we observed that the level of the endogenous BCKDK expression was negligible compared to the level of the exogenous BCKDK expression. Consequently, when HEK-293 cells were transduced at the same time with shRNA to remove the endogenous BCKDK expression and with exogenous BCKDK WT, no difference in total BCKDK expression was observed (Supplementary Figure S1B). The next experiments were then performed without using shRNA for endogenous BCKDK depletion. Western blot visualizing the phosphorylation of the BCKDH E1α-subunit, the natural substrate of BCKDH, shows that the p.Thr334del variant observed in patients significantly reduces the BCKDK capacity to phosphorylate its substrate (Figure 4).

### 2.3. Patients’ Follow-Up and Treatment

The three siblings presented with psychomotor delays in the first year of life. Patients 2 and 3 were reported to have subacute regression during their second year of life, with loss of previously acquired skills. Cranial circumference growth curves showed progressive microcephaly. They did not acquire speech. All presented generalized seizures during early childhood. Patients were treated with a combination of valproate and levetiracetam. Seizures were poorly controlled, especially for patient 2 who was admitted to hospital 10 times in the previous year for prolonged epileptic crises.

**Figure 5:**
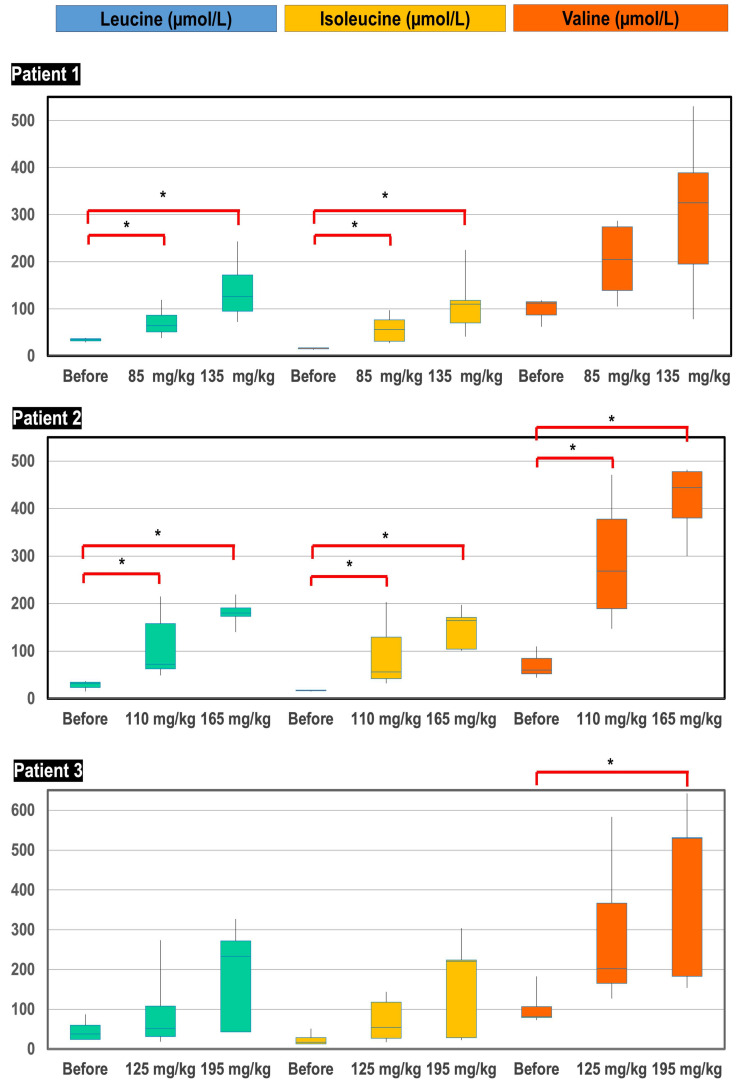
Box-plot distribution of plasma BCAA concentrations (µmol/L) before initiation of treatment, after supplementation of starting doses of BCAA (85, 110 and 125 mg/kg/day for patient 1, 2 and 3, respectively) and after supplementation of high doses of BCAA (135, 165 and 195 mg/kg/day for patient 1, 2 and 3, respectively). * *p*-value < 0.05.

The three patients were treated with a protein-rich diet (2.5–3 g/kg/day) and pharmacological doses of L-leucine, L-isoleucine and L-valine. For practical reasons and due to absence of available data about the pharmacodynamics of BCAA, the three patients received the same dose of BCAA, meaning various weight-related doses. Starting with approximate doses of 85, 110 and 125 mg/kg/day of each amino acid, these were divided four times each for administering in patients 1, 2 and 3, respectively; blood BCAA remained far below the normal range. BCAA supplementation was increased to approximately 135, 165 and 195 mg/kg/day, divided six times, which allowed the obtaining of physiological plasma concentrations (Figure 5). Although pharmacokinetics was not accurately studied, we observed a relationship between plasma BCAA concentrations and timing of sampling after oral intake, suggesting a rapid disposal of BCAA after administration.

**Figure 6:**
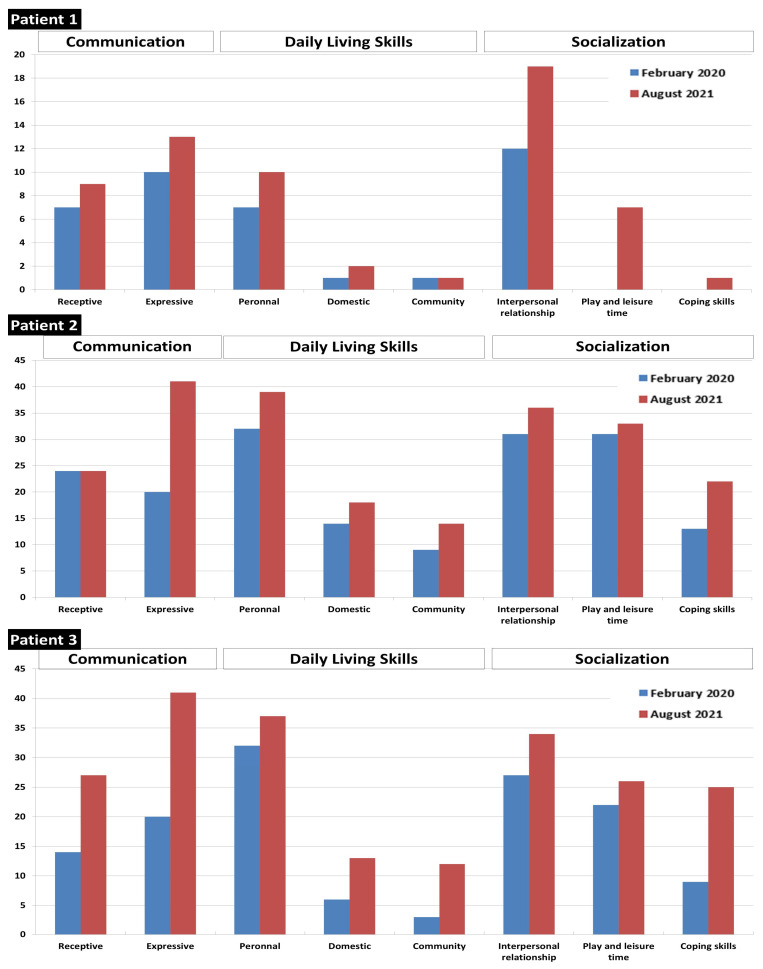
Vineland score before and after 18 months of treatment with high-protein diet and BCAA supplements.

After starting treatment, the parents subjectively noted a clear improvement in the behavior of their children, especially for patient 1 and 3. They did not present any epileptic crises after the start of supplementation. Patient 2, who presented recurrent seizures, was admitted in hospital only once during the 18 months of treatment (compared to 10 admissions in the year preceding BCAA supplementation). The neurodevelopmental skills of patients, assessed by the Vineland Adaptative Behavioral Scale, showed improvement, especially in communication and socialization skills (Figure 6).

## 3. Discussion

This study reports on three patients from an inbred family with BCKDK deficiency. The affected siblings were all carrying the same in-frame c.999_1001delCAC (p.Thr334del) homozygous deletion in the *BCKDK* gene. In order to demonstrate the pathogenicity of this newly described variant, we performed functional studies on cultured transfected cells. We showed that the mutated BCKDK protein was unable to phosphorylate the E1α-subunit of BCKDH complex, leading to an increased consummation of BCAA in the culture media.

BCKDK deficiency is a dietary-treatable disorder of BCAA mitochondrial metabolism [11]. Here, we provide supporting evidence that BCKDK-deficient patients can be managed by a high-protein diet supplemented with BCAA-complements. This therapy improved neurological features of our patients. Nevertheless, steady-state concentrations of BCAA in plasma were challenging to maintain in all three patients, and at least six daily intakes were necessary to correct BCAA levels. Delay between BCAA administration and blood sampling may directly affect the plasmatic concentrations of BCAA. Thus, in order to harmonize BCAA measurements in the course of patients’ follow-up, the pharmacokinetics of BCAA after oral supplementation should be evaluated. Unfortunately, during the present study, we were not able to perform sequential drawing after oral intake to determine the half-life of BCAA in plasma.

Regarding patients’ neurodevelopmental improvement, our results need to be considered with some caution. We acknowledge that the effect of BCAA treatment should be interpreted with regard to the natural history of the disease. BCKDK deficiency is however a very rare inherited metabolic disease; this disorder was described for the first time in 2012 [8] and only a few patients have been reported so far. The natural history of the disease is thus not yet well described, and it is difficult to know whether the patients’ improvement we observed over the 18-month follow-up is a consequence of the treatment or whether it is the “normal” thrive of untreated BCKDK patients. Additionally, the entire phenotypic spectrum of BCKDK deficiency is not currently known and a milder phenotype should exist. We cannot thus predict whether a high-protein diet supplemented with BCAA-complements could benefit all patients with BCKDK deficiency. Further genotype–phenotype correlation studies are therefore needed to demonstrate whether any patients may be managed with such a diet.

Newborn screening mainly focuses on highlighting elevated concentrations of BCAA in order to catch neonates with MSUD. Consequently, low values of BCAA are generally disregarded during the results’ reviewing process. However, the significantly low concentrations of BCAA identified on NBS samples in our patients suggest, that BCKDK deficiency could be identified at birth by using threshold values also emphasizing low BCAA concentrations. Assuming that it would be desirable to prevent rather than trying to reverse symptoms, BCKDK deficiency could therefore be amenable to newborn screening, and prevalence of BCKDK deficiency is suspected to be very low. Moreover, patients with epilepsy are two- to three- times more likely to die prematurely, before the eventual genetic cause has been established [12]. In this context, NBS programs could potentially demonstrate that the disease is underdiagnosed and that intermediate forms eventually exist. Finally, further studies are required to demonstrate whether neonatal screening and early treatment can prevent severe and irreversible neurological damages.

## 4. Materials and Methods

### 4.1. Subjects and Samples

We describe three siblings from a consanguineous family presenting severe encephalopathy, autistic behavior and poorly controlled seizures, in whom exome sequencing was performed to unravel the genetic cause.

Patients were treated during 18 months with a protein-rich diet and BCAA-complements. In the course of follow-up, BCAA supplementation was step-wisely increased to reach physiological plasmatic levels. BCAA concentrations under treatment were compared to initial BCAA levels using the Mann–Whitney test (computed by MedCalc v19.0.5 software).

Diagnostic procedures, follow-up and treatments were carried out with parental consent. Ethical review board approved the management of patients, according to the Declaration of Helsinki (reference 2020/62).

### 4.2. Sequencing

Whole-blood DNA extraction was performed using the NucleoMag^®^ Blood 200 μL kit (Macherey-Nagel, Düren, Germany) automated on a STARlet platform (Hamilton, Reno, NV, USA) following manufacturer’s instructions. Briefly, 200 μL of whole blood were lysed in a buffer containing proteinase K and paramagnetic beads were used to remove contaminants and salt while elution was performed using a low-salt buffer. The quality of isolated genomic DNA was quantified and verified by a Qubit^®^ DNA Assay Kit in a Qubit^®^ 2.0 Flurometer (Invitrogen, Carlsbad, CA, USA) and agarose gel electrophoresis. Whole exome sequencing (WES) was conducted on 2000 ng of genomic DNA from the proband and parents. Fragment libraries were created from the sheared samples. Target enrichment was performed according to the manufacturer’s protocols (Agilent SureSelect Human All Exon V6; Agilent Technologies, Santa Clara, CA, USA), and sequencing was performed on a Novaseq 6000 instrument (paired-end; 300 cycles, with an average depth of coverage of 60×; Illumina, San Diego, CA, USA).

Variants were called and annotated using a homemade bioinformatic pipeline (Humanomics v2.0). Briefly, only regions of the reads with high quality were aligned to the UCSC hg19 reference sequence using BWA-MEM v1.7.17. Variant calling (HaplotypeCaller) and joint genotyping (GenotypeGVCFs) were processed using GATK v3.8. Optical and PCR duplicates were marked and removed with Picard v2.20.

Sanger sequencing was performed to confirm the indel and to validate the segregation of the anomaly within the family. The *BCKDK* gene of the patients and parents was sequenced by Sanger sequencing of the peripheral blood leukocyte DNA. The forward and reverse sequences of the primers for exon 11 of BCKDK are respectively: 5′-GGAAGGAACAGGAAGGCAGACT-3′ and 5′-GGGCAACCCGAAGCCAAAG-3′. Samples underwent standard PCR conditions and were sequenced using a BigDye Terminator kit (Applied Biosystems, Foster City, CA, USA) and run on an ABI 3730xl automated sequencer (Applied Biosystems). SeqScape v.2.6 software (Applied Biosystems) was used to align sequence data against the relevant reference.

### 4.3. Biochemical Analysis

Plasma and cerebrospinal fluid (CSF) analysis of amino acids was performed using a TRAQ kit for amino acid analysis of physiological fluids (Sciex, Framingham, MA, USA), as previously described [13]. Newborn screening (NBS) of amino acids and acylcarnitines was carried out by flow-injection mass spectrometry [14,15]. Amino acids in plasma, CSF and dried blood spots (DBS) were quantified on a TQ5500 mass spectrometer (Sciex).

### 4.4. Lentiviral Constructs

HEK-293 cells depleted for human endogenous BCKDK expression were generated using lentiviral shRNA particles. Pre-designed lentiviral plasmids were obtained from Sigma, Overijse, Belgium: sh_A_CDS = TRCN0000199200, sh_B_CDS = TRCN0000010196, sh_C_CDS = TRCN0000010183, sh_D_CDS = TRCN0000196380 and sh_E_3UTR = TRCN0000199101. The control shRNA was anti-eGFP shRNA plasmid (shRNA anti-eGFP (puro), Sigma No. SHC005). A positive lentiviral plasmid was also purchased to confirm efficient transduction of lentiviral particles: pLKO.1-puro-CMV-TagRFP (puro); Sigma No. SHC012. All plasmids included a puromycin resistance gene for selection of transduced cells. Lentiviral vectors were also used for human wild-type (WT) or mutated BCKDK over-expression. Three different plasmids were designed and purchased through Vector Builder: pLV_EF1a_BCKDK-wt-Flag (mCherry-Hygro) and pLV_EF1a_BCKDK-d334-Flag (mCherry-Hygro). The first plasmid allows the expression of human BCKDK (NM_005881.4) with silent mutations (to avoid shRNA recognition) and 3xFLAG tag under the control of EF1a promoter and eGFP-Blasticidin resistance genes under the control of CMV promoter. The second plasmid allows the expression of human BCKDK with the same silent mutations, mutation p.Thr334del and 3xFLAG tag under the control of EF1a promoter. These two plasmids also allow mCherry and hygromycin resistance genes (mCherry-T2A-Hygromycin) under the control of CMV promoter. We also designed a lentiviral plasmid control in which BCKDK was replaced by EmGFP (pLV_EF1a_EmGFP (Blasti). Lentiviral vectors were produced by GIGA viral vectors platform. Briefly, Lenti-X™ 293T Cell Lines (Clontech, Mountain View, CA, USA; No. 632180) were co-transfected with shRNA or over-expression plasmids for BCKDK, psPAX2 and pVSV-G (Addgene, Cambridge, MA, USA) [16]. Viral supernatants were collected 48–96 h post transfection. HEK-293 cells were transduced for 48 h at a multiplicity of infection (MOI) of 40 when using lentiviral vectors containing the shRNA and their related control, and at MOI of 20 when using the lentiviral vectors containing the BCKDK WT or BCKDK p.Thr334del and their related control.

### 4.5. Cell Culture

HEK-293 cells were grown in DMEM (Westburg, Lesden, The Netherlands) and 10% (*v*/*v*) fetal bovine serum. Three days after lentivirus addition, transduced cells were selected by adding either blasticidin, hygromycin or puromycin depending on the lentivirus used. The cells were prepared at 80% confluence the day before protein extraction and Western blot experiments.

### 4.6. Western Blot

Total protein extraction was performed using a RIPA Lysis and Extraction Buffer (Thermo Scientific, Waltham, MA, USA) supplemented with cOmplete™ Protease Inhibitor Cocktail and PhosSTOP™ (Roche, Basel, Switzerland) phosphatase inhibitor tablets. Total protein extracts (15 µg) were electrophoresed on a 10% SDS-PAGE gel. After transfer and blocking on an Immobilon-P membrane (Millipore, Burlington, MA, USA), the membranes were incubated with the primary antibody, washed, and then incubated with the second peroxidase-conjugated antibody. The reaction was revealed with the Clarity Max ECL kit (Biorad, Hercules, CA, USA). Antibodies used were rabbit anti-Flag (Sigma, Overijse, Belgium; No. F7425) to detect exogenous BCKDK; rabbit anti-Hsp90 (Proteintech, Manchester, UK; No. 13171-1-AP); rabbit anti-BCKDK (Sigma, No. AV52131) to detect both endogenous and exogenous BCKDK; rabbit anti-BCKDH-E1α (E4T3D) (Cell signaling, Danvers, MA, USA; No. 90198); rabbit anti-phospho-BCKDH-E1α (Ser293) (E2V6B) (Cell signaling, No. 40368); and anti-rabbit-IgG HRP-linked (Cell Signaling; No. 7074).

### 4.7. Computed Tertiary Structure

Structural homology between WT and mutant proteins was predicted using Swiss-Model tools (https://swissmodel.expasy.org, accessed on 5 October 2021) [17,18]. Folding deviation between both enzymes was assessed using the Local Distance Difference Test (LDDT), which is a superposition score computing differences in pairwise interatomic distances [19].

## Figures and Tables

**Table 1:** Concentrations (in µmol/L) and ratios of BCAA (with age-related reference values) measured in plasma, CSF and NBS dried blood cards of affected patients and their relatives. Plasma and CSF concentrations of patients are reported at time of diagnosis.

**Patients**		**Plasma BCAA**						**CSF BCAA**						**NBS BCAA**			
**ID**	**Gender**	Leu * (74–203)	Ile * (42–124)	Val * (145–337)	Leu/Phe * (1.2–3.0)	Ile/Phe * (0.7–1.7)	Val/Phe * (2.0–5.4)	Leu * (7–44)	Ile * (4–24)	Val * (10–41)	Leu/Phe * (0.9–2.2)	Ile/Phe * (0.4–1.7)	Val/Phe * (1.1–2.8)	Xle * (<486)	Val * (<269)	Xle/Phe * (<6.0)	Val/Phe * (<3.1)
Patient 1	Female	29	13	62	0.4	0.2	0.9	3.3	1.4	6.4	0.20	0.08	0.38	84	47	1.4	0.8
Patient 2	Female	15	7	44	0.3	0.1	0.9	1.8	1.0	4.4	0.11	0.06	0.28	89	44	1.8	0.9
Patient 3	Male	51	21	73	0.6	0.3	0.9	3.8	1.4	6	0.23	0.08	0.36	210	81	3.2	1.2
**Unaffected Siblings**		**Plasma BCAA**						**CSF BCAA**						**NBS BCAA**			
**ID**	**Gender**	Leu * (74–203)	Ile * (42–124)	Val * (145–337)	Leu/Phe * (1.2–3.0)	Ile/Phe * (0.7–1.7)	Val/Phe * (2.0–5.4)	Leu * (7–44)	Ile * (4–24)	Val * (10–41)	Leu/Phe * (0.9–2.2)	Ile/Phe * (0.4–1.7)	Val/Phe * (1.1–2.8)	Xle * (<486)	Val * (<269)	Xle/Phe * (<6.0)	Val/Phe * (<3.1)
Sister 1	Female	126	62	177	1.3	0.7	1.9	/	/	/	/	/	/	321	145	5.7	2.6
Brother 1	Male	125	58	136	2.0	0.9	2.2	/	/	/	/	/	/	246	112	4.4	2.0
Sister 2	Female	112	46	130	2.3	1.0	2.7	/	/	/	/	/	/	253	81	4.8	1.5
**Parents**		**Plasma BCAA**															
**ID**	**Gender**	Leu * (74–228)	Ile * (37–132)	Val * (105–352)	Leu/Phe * (0.9–2.6)	Ile/Phe * (0.7–1.5)	Val/Phe * (1.7–4.8)										
Father	Male	156	66	222	2.4	1.0	3.4										
Mother	Female	134	64	175	1.6	0.8	2.1										

* Leu, Ile, Val and Xle stand for leucine, isoleucine, valine and leucine/isoleucine respectively. Leu/Phe, Ile/Phe and Val/Phe are the corresponding ratios calculated on respective phenylalanine concentrations.

## Data Availability

The datasets used and/or analyzed during the current study are available from the corresponding author on reasonable request.

## Supplementary Materials

The following supporting information can be downloaded at: https://www.mdpi.com/article/10.3390/ijms23042253/s1.

## Abbreviations

BCKDK	Branched-Chain α-Ketoacid Dehydrogenase Kinase
BCKDH	Branched-Chain α-Ketoacid Dehydrogenase
BCAA	Branched-Chain Amino Acids
CSF	Cerebrospinal Fluid
DBS	Dried Blood Spots
NBS	Newborn Screening
WES	Whole Exome Sequencing
