# Supplementary material for: Novel Loss of Function Variant in BCKDK Causes a Treatable Developmental and Epileptic Encephalopathy
Source: Int J Mol Sci. 2022 Feb 18;23(4):2253. doi: 10.3390/ijms23042253 (PMC8878489; doi:10.3390/ijms23042253)
Supplement: Supplementary file 1 [file ijms-23-02253-s001.zip › BCKDK_Suppl_Fig1.pdf]

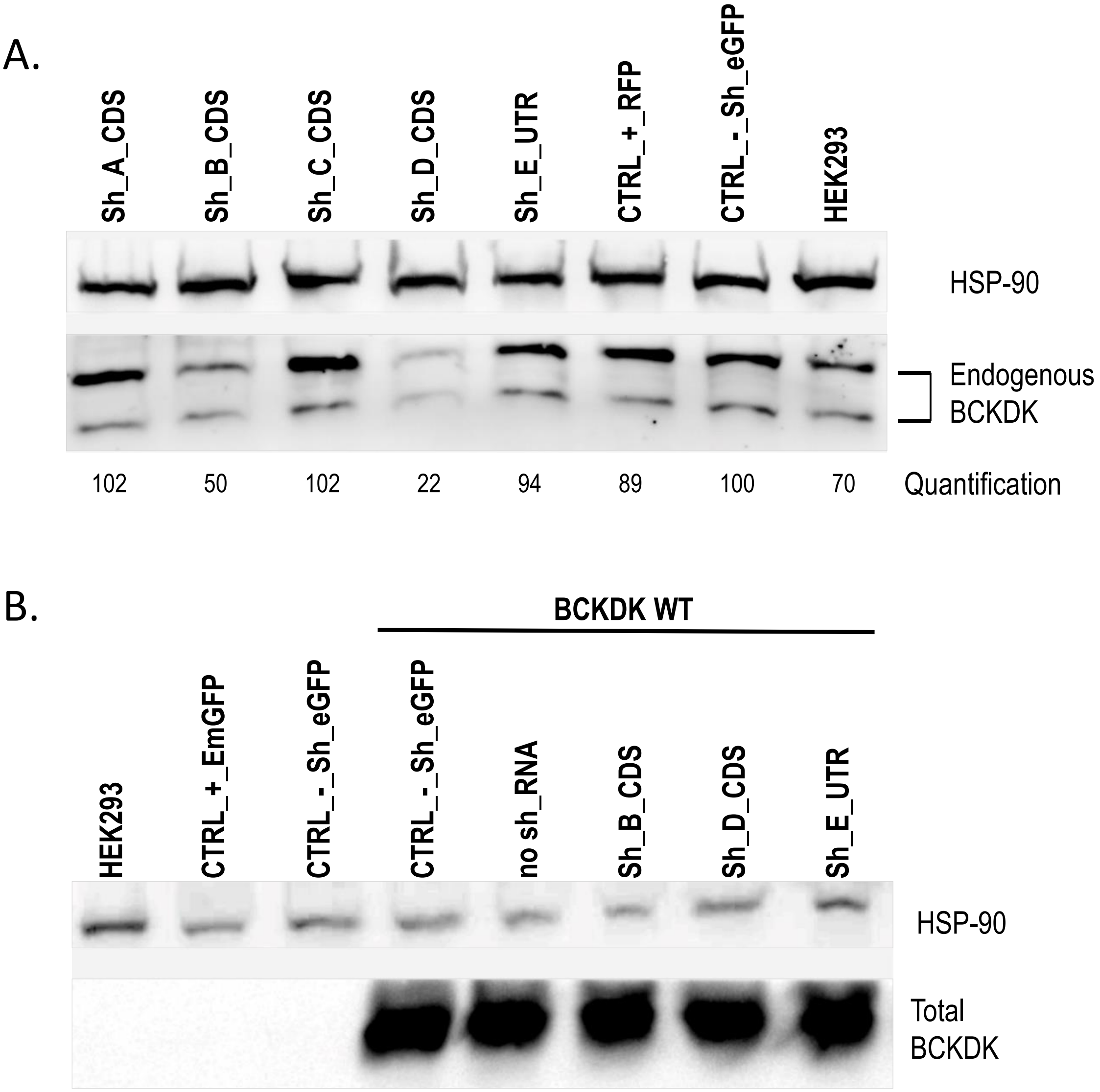

Supplemental Figure 1 :
Western blot to detect endogenous BCKDK (Ab anti-BCKDK) or HSP90 as charge control. A. From left to right: HEK293 cells were transduced using 5 different Sh RNA directed against endogenous BCKDK, a transduction control (lentivirus containing RFP mRNA), a sh RNA directed against an unrelated target (eGFP) or left untreated. B. From left to right: HEK293 were left untreated, transduced with a transduction control (EmGFP), or a sh RNA directed against an unrelated target (eGFP) ; HEK293 were simultaneously transduced with wild type exogenous BCKDK and a lentivirus containing a shRNA directed against an unrelated target (eGFP) ; HEK293 cells were transduced with exogenous wild type BCKDK only, HEK293 were simultaneously transduced with wild type exogenous BCKDK and lentivirus containing 3 different shRNA directed against wild type endogenous BCKDK
